# High-Grade Primary Spindle Cell Sarcoma of the Prostate: A Case Report and Review of the Literature

**DOI:** 10.4021/wjon757w

**Published:** 2014-01-16

**Authors:** Hakan Ozturk, Oya Nermin Sivrikoz

**Affiliations:** a Department of Urology, Sifa University School of Medicine, Izmir, Turkey; b Department of Pathology, Sifa University School of Medicine, Izmir, Turkey

**Keywords:** Spindle cell sarcoma, Prostate, Sarcoma

## Abstract

Evaluation of the primary spindle cell sarcoma prostate, which is seen extremely rare, is aimed. Literature search was made through a search in the MedLine database using PubMed and Scopus for the articles published between January 1988 and September 2013. Electronic search was limited to the following keywords: “spindle cell sarcoma”, “primary prostate sarcoma”. Primary prosatate sarcoma was reported as 100 patients in literature. High-grade primary spindle cell sarcoma of the prostate was encountered as 16 patients in literature. This patients was reported as the 17th case. It is a difficult cancer due to diagnosis and treatment because of the short average life expectancy, high potential of asymptomatic aggressive and metastatic. Histopatogenesis is not exactly known. In 50% of composed of patients who are previously diagnosed with prostate adenocarcinom. The case was reported as high-grade primary prosatate sarcoma at an early age. The case is different from other cases because of not being adenocarcinom component.

## Introduction

Prostatic sarcomas made 0.1 to 0.2% of all malignant prostate tumors. Rhabdomyosarcoma is frequent during childhood whereas leiomyosarcoma is more frequent in adults [1]. In the literature, the number of the cases with diagnosed primary prostatic sarcoma is 100 [2]. In the past, stromal tumors of the prostate were reported using several terms including atypical stromal hyperplasia. Currently, these tumors are classified according to WHO classification as follows: prostatic sarcomas, stromal tumors of unknown malignant potential and stromal prostatic sarcoma (high- and low-grade) [3]. Our patient was reported as having high-grade spindle cell prostatic sarcoma. In the current literature, high-grade spindle cell prostatic sarcoma has been reported in only 16 patients [4, 5]. Clinical signs of the disease include low urinary tract symptoms (LUTSs). The patient rarely has hematuria, painful voiding and rectal pain [6].

## Case Report

**Figure 1:**
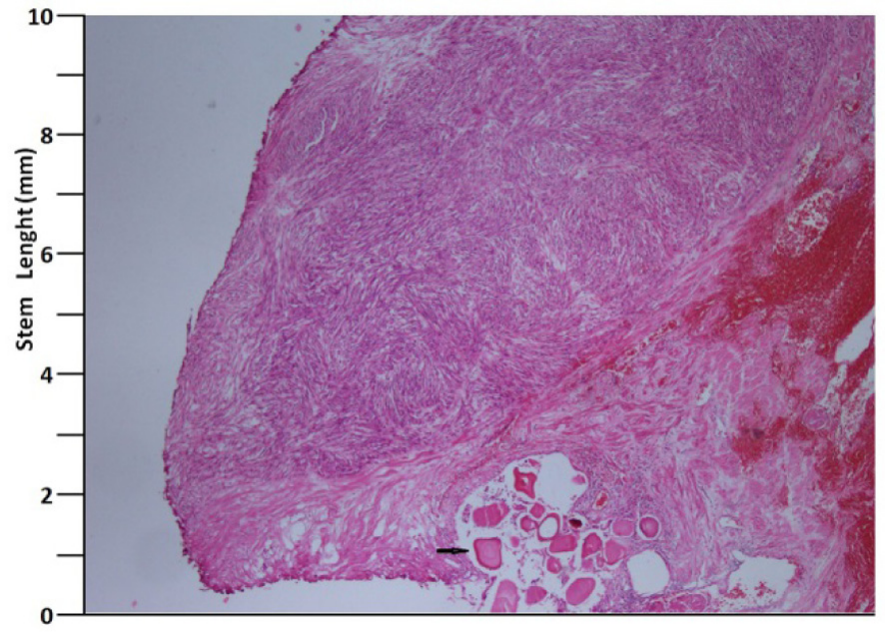
H&E, × 40 (arrow: corpora amilacea).

**Figure 2:**
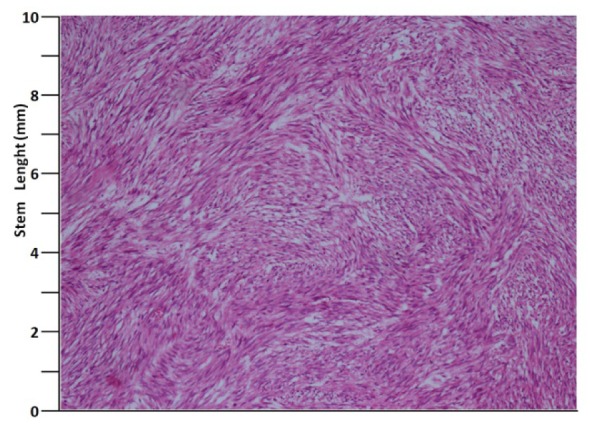
H&E, × 100.

**Figure 3:**
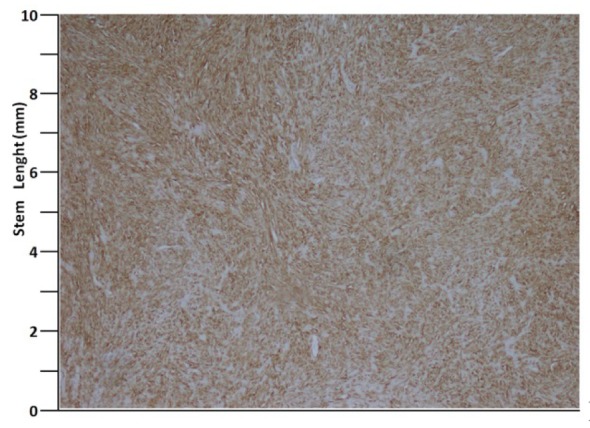
Actin, × 40.

A 41 years old man presented with low urinary tract symptoms lasting for 8 months and progressing steadily. Percutaneous cystostomy catheter was inserted to the patient who could not be catheterized via transurethral route. His laboratory investigations were as follows: hepatic function tests and alkali phosphatase normal; urea: 51 mg/dL; creatinine: 1.48 mg/dL; complete urinary analysis: plenty of erythrocytes and 3 - 4 leukocytes per field; no growth in the urine culture. PSA was measured as 0.7 ng/mL. The prostate was palpated as diffusely hard in the digital rectal examination. The patient underwent palliative transurethral prostatic resection. On the endoscopic view, the crista urethralis, verru montanum and prostatic urethra had irregular anatomy and the prostatic urethra appeared to be obliterated due to presence of tumor. Macroscopically, 25 cc of tissue was resected. Pathologic examination revealed high-grade spindle cell sarcoma of the prostate and widespread mitosis and necrosis in 8 of 10 magnification fields (Fig. 1, 2). In immunohistochemical examination presence of actin, vimentin, desmin, CD34, S100 and PR was examined. Diagnosis of spindle cell leiomyosarcoma was made (Fig. 3). PET/BT scanning revealed widespread lung metastases. Adjuvant chemotherapy was scheduled.

## Discussion

Spindle cell lesions of the prostate are included in a broad spectrum covering both benignant and malignant processes. In the differential diagnosis, one should consider many benignant and malignant tumors such as some leiomyomas and leiomyosarcomas, rhabdomyosarcoma, inflammatory myofibroblastic tumor, solitary fibrous tumors and phylloides tumor [7]. Spindle cell sarcoma of the prostate is one of the rare, insidiously progressing, aggressive variant tumors with high potential to make metastasis. More than half of the patients diagnosed as having these tumors consist of the cases diagnosed as having prostatic adenocarcinoma and showing sarcomatoid differentiation as a consequence of the treatments. This differentiation occurs over a long time period ranging from 6 months to 16 years. In the literature, average time of differentiation is 7 years [8].

Histopathogenesis of the spindle cell cancer of the prostate is not exactly known. Proposed mechanisms include transformation of the epithelial structures to sarcomatous components and two-way differentiation of the epithelial stem cells to both malignant components [9]. According to these theories, the disease process may occur through two separate differentiation pathways. The more commonly accepted theory, however, is that the disease arises from one origin and it creates a different form with sarcomatoid differentiation. Operation materials and specimens of these patients obtained during diagnostic procedures for sarcomatoid carcinoma also indicate that the simultaneous carcinomas contain high-grade epithelial components [10]. Thus, sarcomatoid differentiation is more common in the presence of high-grade tumor. Receiving therapies such as hormonal therapy and/or radiation therapy is considered to trigger sarcomatoid differentiation [10].

One-year mortality of the disease is 20% [10]. Average survival is 9.5 - 14 months. Survival is directly related especially to stage of the tumor and presence of metastasis. Presence of necrosis, grade of the tumor and presence of adenocancer component are not parameters affecting survival directly. Difficulty of diagnosis and treatment of this disease is related to the fact that these malignant tumors do not produce PSA and no other biochemical parameter exists indicative of them. Furthermore, short time to progress and the LUTSs are only seen in the advanced cases cause difficulty in diagnosis. The fact that they are included in the rare causes of the LUTS prolongs the time to diagnosis and diagnosis is possible only by means of biopsy. Additionally, the fact that the disease begins as adenocarcinoma and inability to detect the variant components in the first biopsy are other difficulties in the diagnosis. The fact that serial PSA measurement is not conclusive in the patients receiving androgen blocking treatment with diagnosis of prostatic adenocarcinoma is also an important issue. In the patient presented here, the disease process began primarily without adenocarcinoma component in contrast to majority of the cases in the literature and at the time of diagnosis the patient had widespread metastases in the lungs, which are not primary metastatic site for the prostatic carcinoma. Treatment of the local disease is with curative methods such as radical prostatectomy. But the disease has high potential to generate locally advanced disease and metastases. Chemotherapies used in treatment of the sarcomas are almost the first option and may be combined with radiation therapy.

In conclusion, spindle cell sarcoma of the prostate is a disease very hard to diagnose and treat. Multiplicity of the metastatic cases at the time of diagnosis limits therapeutic options.

Long-term follow-up is usually impossible due to poor prognosis and potential to make metastasis. Impact of the adjuvant or neo-adjuvant chemotherapy and the radiation therapy remains limited because of poor differentiation of the tumor. Option of surgical treatment is possible only for local disease [10].
